# The effect of rapamycin and its analogues on age-related musculoskeletal diseases: a systematic review

**DOI:** 10.1007/s40520-022-02190-0

**Published:** 2022-07-21

**Authors:** Hong Lin, Felipe Salech, Anthony Lim, Sara Vogrin, Gustavo Duque

**Affiliations:** 1 Australian Institute for Musculoskeletal Science (AIMSS), Geroscience and Osteosarcopenia Research Program, The University of Melbourne and Western Health, VIC St. Albans, Australia; 2 Department of Medicine - Western Health, The University of Melbourne, VIC St Albans, Australia; 3 Melbourne Medical School, The University of Melbourne, St Albans, VIC, Australia; 4 Sección de Geriatría, Clínica de Caídas Y Fracturas, Hospital Clínico Universidad de Chile, Santiago, Chile; 5 Centro de Investigación Clínica Avanzada (CICA), Hospital Clínico Universidad de Chile, Santiago, Chile; 6 Centro de Gerociencia, Salud Mental Y Metabolismo (GERO), Santiago, Chile

**Keywords:** mTOR, Osteosarcopenia, Rapamycin, Rapalogues, Rheumatoid arthritis, Osteoarthritis

## Abstract

**Background:**

Preclinical studies have shown a therapeutic role of the mechanistic/mammalian target of rapamycin complex 1 (mTORC1) inhibition with rapamycin and its analogues (rapalogues) on several age-related musculoskeletal disorders (MSKD). However, the applicability to humans of these findings is unknown.

**Objective:**

To assess the efficacy of rapalogues on age-related MSKD in humans.

**Methods:**

We conducted a systematic review according to the PRISMA guidelines. MEDLINE, EMBase, EMCare, and Cochrane Central Registry of Controlled Trials were searched for original studies examining the effects of rapalogues on outcomes linked to the age-related MSKD in humans. This review is registered in the PROSPERO database (University of New York; registration number CRD42020208167).

**Results:**

Fourteen studies met the inclusion criteria and were analyzed. The effect of rapamycin and other rapalogues, including everolimus and temsirolimus, on bone, muscle and joints have been evaluated in humans; however, considerable variability concerning the subjects’ age, inclusion criteria, and drug administration protocols was identified. In bone, the use of rapamycin is associated with a decrease in bone resorption markers dependent on osteoclastic activity. In muscle, rapamycin and rapalogues are associated with a reduction in muscle protein synthesis in response to exercise. In the context of rheumatoid arthritis, rapamycin and rapalogues have been associated with clinical improvement and a decrease in inflammatory activity.

**Conclusion:**

Although there are studies that have evaluated the effect of rapamycin and rapalogues on MSKD in humans, the evidence supporting its use is still incipient, and the clinical implication of these results on the development of osteoporosis, sarcopenia, or osteosarcopenia has not been studied, opening an interesting field for future research.

## Introduction

Musculoskeletal disorders (MSKD) affect bones, joints, ligaments, and muscles and are among the most common health problems in older people, affecting up to 80% of people over 65 [1]. MSKD in older adults are associated with disability [2], loss of independence, poor quality of life [3], mortality [4], and high healthcare costs [5]. The annual cost of MSKD is approximately 1 billion British pounds in the UK, and over 80 billion euros globally [5]. The most prevalent MSKD in older persons are osteoarthritis, osteoporosis (bone loss that predisposes to fractures), inflammatory arthritis (i.e., rheumatoid arthritis (RA)) and sarcopenia (loss of muscle mass, strength and/or function) [1]. Some MSKD can coexist in the same patient enhancing their harmful effects on health. Osteosarcopenia is a well-defined syndrome of concurrent osteoporosis and sarcopenia, contributing to adverse outcomes and reduced functional capacity [6, 7]. Over 144,000 osteoporotic fractures are reported yearly, while 40% of the ‘high-risk’ population suffering prior falls also presented with osteosarcopenia [8].

Currently, MSKD treatment involves medications and non-pharmacological (i.e., exercise and nutrition) interventions. However, available medications for osteoporosis and RA are limited by side effects, highly prevalent in older adults, and although non-pharmacological interventions, such as exercise, are effective in sarcopenia and osteoarthritis, they are affected by reduced adherence and baseline function [9]; thus, the search for novel therapeutic alternatives is mandatory.

The close relationship between MSKD and age suggests that common mechanisms between the biology of aging and the pathophysiology of MSKD may exist. In this sense, emerging anti-aging therapies could have a therapeutic role in managing MSKD. The mammalian target of rapamycin (mTOR) is a kinase that regulates several cellular aging processes, including cell growth, translation, and autophagy [10–12]. The inhibition of mTOR activity, by genetics or through pharmacological interventions, has increased maximal lifespan and health span in several animal species and is currently one of the most studied anti-aging interventions. Rapamycin, a natural macrocyclic lactone produced by the bacterium *Streptomyces hygroscopicus*, is a well-known mTOR inhibitor that can be administered in humans. Rapamycin binds to the immunophilin FK Binding Protein-12 (FKBP-12) in mammalian cells to generate a complex that binds to and inhibits mTOR activation [13]. Analogues of rapamycin (“rapalogues”) have been developed to optimize the pharmacokinetics of rapamycin-mediated mTOR inhibition leading to more favourable clinical outcomes [14, 15].

Preclinical data has demonstrated that rapamycin or rapalogue-induced mTOR inhibition protects against MSKD. Luo et al. [16] administered rapamycin to 24-month-old rats, resulting in increased trabecular bone mineralization associated with declining osteoclasts and elevated autophagy activity in osteocytes. Other studies in mice also show that the use of rapamycin or rapalogues ameliorates age-related muscle atrophy [17], whereas chronic mTOR activity leads to a decline in skeletal muscle mass [18]. These studies support targeting mTOR activity as a potential treatment for osteoporosis, sarcopenia, or osteosarcopenia. In other murine models of osteoarthritis, targeting mTOR activity with rapamycin delays or reduces joint cartilage degradation [19, 20]. In addition, rapamycin’s immunosuppressive effects have been used with clinical efficacy in cancer patients (e.g., renal cell carcinoma) [13], supporting rapamycin’s anti-inflammatory action and potential as a treatment for inflammatory MSKD (e.g., RA).

The consistency of improved outcomes following targeting mTOR activity across experimental models of MSKD calls for an investigation of the usefulness of rapamycin and rapalogues for treating chronic age-related MSKD in humans. Therefore, this systematic review aims to identify clinical studies using rapamycin and rapalogues to better understand their effects on the musculoskeletal system and address their potential therapeutic value.

## Methods

### Search strategy

This review was registered at PROSPERO (University of York) with registration number CRD42020208167 and conducted according to the Preferred Reporting Items for Systematic Review and Meta-Analyses (PRISMA). The search included MEDLINE, EMBASE, EMCARE, and the Cochrane Controlled Register of Trials. Results comprised papers available from September 2021 to the inception of the database. Key terms for interventions included the following: Rapamycin, rapalog*, Sirolimus, Everolimus, Temsirolimus, Ridaforolimus, Deforolimus and Zotarolimus. Terms used for conditions of interest included: muscle atrophy, sarcopenia, osteoporosis, bone disease, bone erosion, bone fragility, osteosarcopenia, demineralization, metabolic bone, osteoarthritis, and rheumatoid arthritis. The search also included terms that the MeSH dictionary may miss. EMBASE and EMCARE were limited to only articles and articles in press for relevant study types. An example of the search is shown in Table 1.

**Table 1:** Example search strategy on EMBase (via Ovid)

No.	Searches	Results
1	Muscle atrophy/	36,116
2	Sarcopenia/	10,943
3	(sarcop?en* or osteosarcop?en*).ti,ab,kw	14,877
4	("age-related musc*" or "ag?ing musc*" or "muscle wast*" or "muscle loss").ti,ab,kw	10,775
5	("skeletal muscle*" or "muscle mass").ti,kw	69,777
6	Osteoporosis/	121,817
7	Bone disease/ or bone erosion/ or bone fragility/ or demineralization/ or metabolic bone disease/	47,356
8	Osteoarthritis/	93,622
9	Rheumatoid arthritis/	201,956
10	("bone loss" or "ag?ing bone*" or "age-related bone*" or "bone mineral density" or BMD or "bone density").ti,ab	112,175
11	(sirolimus or everolimus or temsirolimus or ridaforolimus or deforolimus or zotarolimus).ti,ab	29,364
12	rapamycin.ti,kw	11,712
13	rapalog*.ti,ab,kw	604
14	1 or 2 or 3 or 4 or 5 or 6 or 7 or 8 or 9 or 10	603,025
15	11 or 12 or 13	40,098
16	14 and 15	589
17	Limit 16 to (article or article in press)	333

### Inclusion and exclusion criteria

Inclusion criteria:

- Rapamycin and rapalogues used as interventions could include:
  - Sirolimus (rapamycin)
  - Temsirolimus
  - Everolimus
  - Ridaforolimus (deforolimus)
  - Zotarolimus
- Age-related MSKD in the study should relate to either:
  - Bone mineral disorders
  - Skeletal muscle loss
  - Osteoarthritis
  - RA
- Randomized control trials (RCT) or controlled clinical trials (CCT) that compared the use of the rapalogue/rapamycin, either as a monotherapy, combination therapy, or adjuvant, to the current standard treatment or placebo for the condition of interest.
- Other types of trials or study types (e.g., single-arm clinical trials or cross-sectional studies) should include the indicated interventions either as monotherapy, combination therapy, or adjuvant as part of the intervention/exposure and evaluate their effects on the conditions of interest.
- The intervention outcomes on the condition of interest should have the processes of measurement explained. This may include (depending on the study) physician assessment, imaging, bone density scans, blood markers, quality of life, mobility, records of falls and/or fractures, changes in weight, adverse events, and muscle mass or function changes.
- Participants aged 18 or over.
- Study in English or translated to English.
- No restriction on publication date.

Exclusion criteria:

- Animal or in vitro studies.
- Case reports or reviews.
- Outcome measure inappropriate (e.g., lacking information on how it was measured, only a summary of outcomes with no supporting information).

### Data extraction

Both COVIDENCE and Google Sheets recorded the outcomes of risk assessment and relevant study data of each paper. Tools used for assessing the risk of bias included the Cochrane RoB 2.0 [21] and ROBINS-I [22]. The assessments were initially conducted independently by Hong Lin and Anthony Lim. Both assessors then discussed the outcomes to reach a consensus.

## Results

**Fig. 1:**
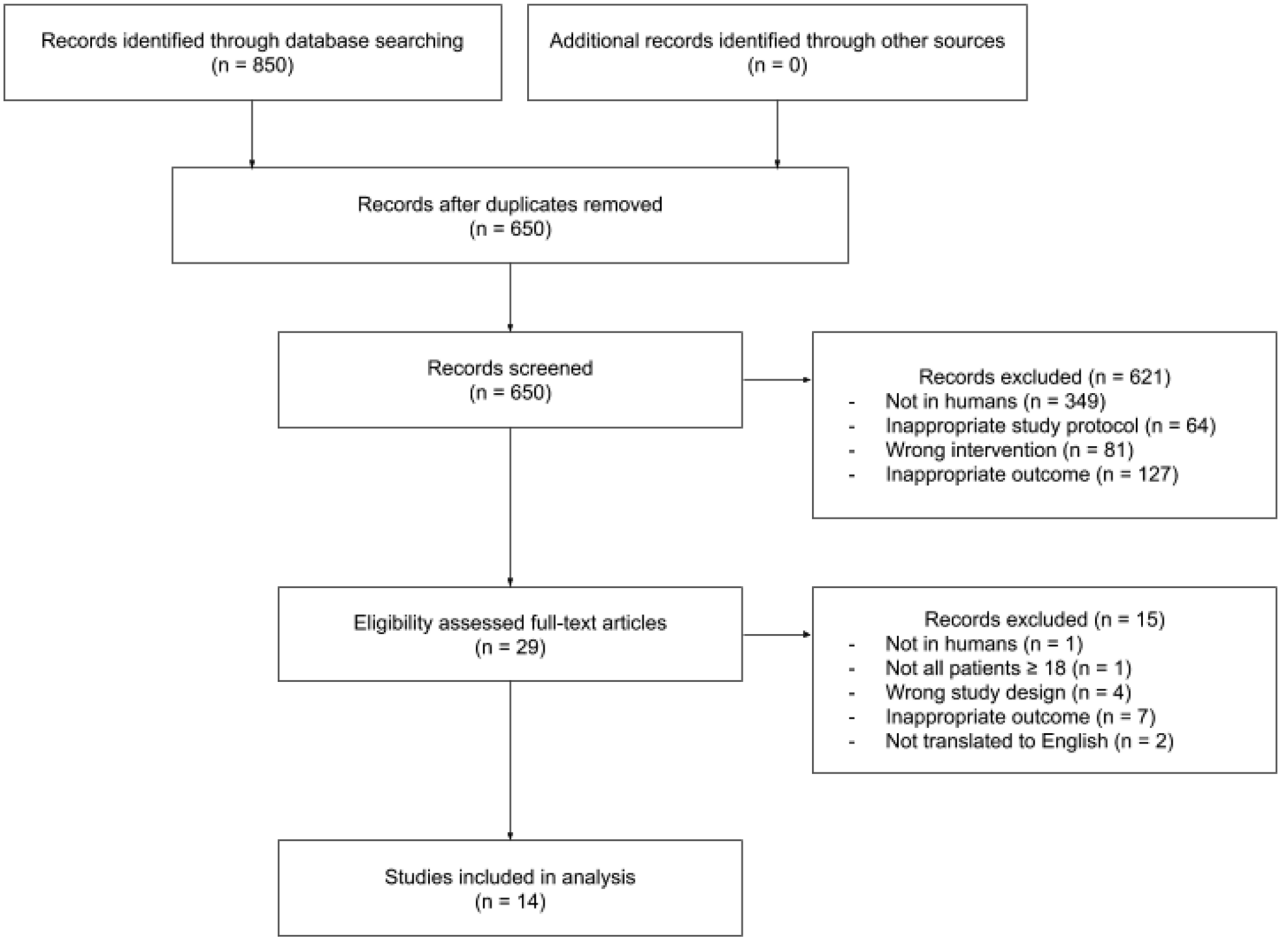
Search Strategy flow chart

The literature search produced 850 results in total (Fig. 1). Six hundred and fifty results were retrieved for screening after removing duplicates (*n* = 200). After title/abstract screening and full-text review, fourteen studies were obtained for review. The main reasons for exclusion included: non-human study, participants under 18, inappropriate study protocol (incorrect or inconsistent study design), incorrect intervention, irrelevant outcomes, and non-English language. The search strategy flow chart is shown in Fig. 1

### Risk of bias assessment

Risk of bias was assessed for all studies included in this review and is shown in Table 2.

**Table 2:** Risk of bias assessment for all studies included in the review

Study	Risk of bias judgment	Comments
Gundermann et al. [28] Activation of mTORC1 signalling and protein synthesis in human muscle following blood flow restriction exercise is inhibited by rapamycin	Low (Cochrane RoB 2.0)	- Well-described method including time points of rapamycin administration vs. tracer material infusion, muscle biopsy and blood sampling times. Important given the time to reach peak concentration of rapamycin - No difference in measured parameters of phospho-mTOR, phospho-S6K1, phospho-Erk1/2, phospho-Mnk1 at baseline - Authors note limitations with providing a dosage that guarantees complete inhibition of mTORC1: dose used was much lower than that often used in animal (rodent) experiments
Dickinson et al. [29] Mammalian target of rapamycin complex 1 activation is required for the stimulation of human skeletal muscle protein synthesis by essential amino acids	Low (Cochrane RoB 2.0)	- No variation in administration of the essential amino acid solution between rapamycin and control groups - No difference in measured parameter of mixed muscle protein fractional synthesis rate at baseline (i.e., pre-essential amino acid solution administration)
Dickinson et al. [30] Rapamycin does not affect post-absorptive protein metabolism in human skeletal muscle	Low (Cochrane RoB 2.0)	- No difference in measured parameters of mixed muscle fractional synthesis rate and whole-body phenylalanine rate of appearance at baseline - Authors note limitations of analyzing basal protein metabolism later course, as study period only included the first 2 h post-rapamycin ingestion (i.e., close to the peak circulating level of rapamycin post-administration)
Drummond et al. [31] Rapamycin administration in humans blocks the contraction-induced increase in skeletal muscle protein synthesis	Low (Cochrane RoB 2.0)	- No difference in measured parameters of serum hormones (cortisol, insulin), amino acids (leucine, isoleucine, valine, phenylalanine), mixed muscle protein fractional synthesis rate at baseline - Authors note limitations with providing a dosage that guarantees complete inhibition of phosphorylation of mTOR, S6K1, rpS6 pre-exercise: dose used was much lower than that often used in animal experiments
Veasey-Rodrigues et al. [32] A pilot study of Temsirolimus and body composition	Serious (ROBINS-I)	- Participants were almost exclusively below ECOG 2 (*n* = 15/16), indicating relatively good baseline function - Participants had a wide age range (36–71) and various types of cancers. The cancer diagnoses distribution is documented and appears skewed toward cancers that occur exclusively in females (25% endometrial, 18% ovarian, 12% cervical) - Grouping patients based on toxicity status and allowing dose reductions and treatment interruptions introduce possible confounding. Notably, there does not appear to be a protocol to describe the exact dose reduction and the frequency of treatment interruptions
Gyawali et al. [33] Muscle wasting associated with the long-term use of mTOR inhibitors	Serious (ROBINS-I)	- Study includes primarily only one cancer (90% renal cell carcinoma) in the study population, making the study participants relatively comparable - Study eligibility did not adequately address many other confounders contributing to muscle wasting. Regarding dosage of intervention, dose-skipping was allowed due to side effects. Notably, the dose of everolimus and Temsirolimus may have differed among participants, but this information was not documented - Study did not document many other diseases or groups of diseases for exclusion that may influence muscle wasting
Campistol et al. [23] Bone metabolism in renal transplant patients treated with cyclosporine or sirolimus	High (Cochrane RoB 2.0)	- No variation in administration of glucocorticoids between sirolimus and control (cyclosporine) groups. This resulted in no difference in cumulative glucocorticoid dose between groups - Open-label design introduces bias - Dose of sirolimus was carefully titrated to achieve chosen steady-state concentration for each intervention period - High dropout rate ~ 28% (161 enrolled, but analysis only includes 115 patients) - Potential omission of data when discussing individual vs. pooled analyses of studies. The author states “the same pattern of differences between the groups was observed…(data not shown).”
Westenfeld et al. [24] Impact of sirolimus, tacrolimus and mycophenolate mofetil on osteoclastogenesis—implications for post-transplantation bone disease	Moderate (ROBINS-I)	- No differences in characteristics of kidney transplant patients at baseline - Intervention was commenced at different times between participants, resulting in varying treatment durations. An average/median time to treatment is not documented - Authors note that patients treated with sirolimus might be more likely to take a lower cumulative steroid dosage, i.e., the presence of another intervention (steroids) is different at baseline between sirolimus vs. control groups - Baseline confounding was also possible due to the large differences in the meantime from transplantation, i.e., different states of osteodystrophy depending on time from transplant and follow-up investigation and treatment since]
Sessa et al. [25] Immunosuppressive agents and bone disease in renal transplant patients with hypercalcemia	Moderate (ROBINS-I)	- Appropriate use of exclusion criteria to minimize confounders of co-morbid disease, particularly chronic inflammatory disorders, smoking, and excessive alcohol intake, all of which may contribute to bone disease - 5 groups were created, each with different immunosuppressive protocols. Notably, the mean age of each group is appreciably different. On age alone as a confounder, this would already make a comparison between groups complexes - Elaboration on whether certain medications were restricted was required to strengthen the study - It is unclear whether the start and follow up times of the intervention/study differed between participants/groups
Gnant et al. [26] Effect of Everolimus on Bone Marker Levels and Progressive Disease in Bone in BOLERO-2	High (Cochrane RoB 2.0)	- No major dropout rate in the full analysis set (ITT), but major dropout rates in the safety analysis set: 404/482 discontinued intervention in the everolimus group, 229/238 discontinued intervention in the placebo group. While the safety analysis set is not the basis for the conclusions in the study, the high dropout rates are noteworthy. The sirolimus group contains ~ 102% more participants for analysis vs. the placebo group - No differences in characteristics in bone metastases and bisphosphonate use at baseline - Possible detection bias where local investigators could order additional scans or surveys at their discretion - Sponsored by Novartis, a major pharmaceutical company
Hadji et al. [27] The impact of mammalian target of rapamycin inhibition on bone health in postmenopausal women with hormone receptor-positive advanced breast cancer receiving everolimus plus exemestane in the phase IIIb 4EVER trial	Moderate (ROBINS-I)	- No difference in measured parameters of CTX, osteocalcin, P1NP, PTH, 25-OH-vitamin D at baseline between groups - Study design allowed anti-resorptive therapy to be used among participants. Although well-addressed in the analysis, the participants received different treatment dosages, negatively impacting the analyses
Bryun et al. [34] Everolimus in patients with rheumatoid arthritis receiving concomitant methotrexate: a 3-month, double-blind, randomized, placebo-controlled, parallel-group, proof-of-concept study	High (Cochrane RoB 2.0)	- No difference in measured parameters of a tender or swollen joint count, pain, patient, physician global disease activity, HAQ physical function, ESR or CRP - Some concerns with subjectivity in measurement, e.g., visual pain scale - Unclear justification in the grouping of some results, i.e., those that dropped out early grouped with those that used steroids - The average cumulative dose of systemic steroids over the 12-week treatment period was not significantly different, but dose adjustments were also allowed at investigators' discretion, given specific blood results
Wen et al. [35] Low-dose sirolimus immunoregulation therapy in patients with active rheumatoid arthritis: a 24-week follow-up of the randomized, open-label, parallel-controlled trial	High (Cochrane RoB 2.0)	- Sirolimus group contains 110% more participants for analysis vs. the conventional group. Importantly, concurrent immunosuppressive agents appear similar in proportion between groups - Open-label design introduces bias - Participants were able to freely use other use prednisone and other immunosuppressive medications to meet the treat-to-target recommendations
Niu et al. [36] Sirolimus selectively increases circulating Treg cell numbers and restores the Th17/Treg balance in rheumatoid arthritis patients with low disease activity or in DAS28 remission who previously received conventional disease-modifying anti-rheumatic drugs	Serious (ROBINS-I)	- Appropriate use of a third group—the “healthy” group, which comprised participants with no known co-morbidities or immunosuppressive use. This appears to serve as an appropriate baseline for Treg cell numbers measured by flow cytometry - No difference in characteristics of rheumatoid arthritis patients at baseline. Importantly, concomitant immunosuppressive agents appear similar in proportion between rheumatoid arthritis patients treated with sirolimus vs. conventional treatment - Confounding was possible given how other immunosuppressive medications were allowed. In addition, the reviewing physician could freely change the dosages of these medications during the study

### Main outcomes

This systematic review aims to provide insights into the effect/s of rapamycin and rapalogues on the most prevalent age-related MSKD in human participants. Some studies measured multiple outcomes, so we focused on measurements/outcomes relevant to the review. Study characteristics are summarized in Table 3.

**Table 3:** Study characteristics

Authors	Year	Study type	Participant characteristics	Sample size	Relevant Outcome(s)	Intervention(s)	Comparison	Duration
Gundermann et al. [28]	2014	RCT	Healthy, recreationally active males	16	Skeletal muscle maintenance/loss	Sirolimus, 16 mg, once 1 h prior to exercise	Control	2 days
Dickinson et al. [29]	2011	RCT	Healthy, recreationally active	8	Skeletal muscle protein synthesis	Sirolimus, 16 mg, once 2 h following 1st muscle biopsy	Control	4 days (2 days for 2 trials)
Dickinson et al. [30]	2013	RCT	Healthy young males and females	6	Skeletal muscle maintenance/loss	Sirolimus, 16 mg, 2 h following 1st muscle biopsy	Control	4 days (2 days for 2 trials)
Drummond et al. [31]	2009	RCT	Healthy young males	15	Skeletal muscle maintenance/loss	Sirolimus, 12 mg, once 1 h after 1st muscle biopsy	Control	2 days
Veasey-Rodrigues et al. [32]	2013	Non-randomized clinical trial	Had documented advanced solid tumors	26	Skeletal muscle maintenance/loss	Temsirolimus, 25 mg IV, weekly	Baseline	8 weeks
Gyawali et al. [33]	2016	Cohort (retrospective)	Patients who had taken everolimus or Temsirolimus for at least 6 months as single drug therapy	20	Skeletal muscle maintenance/loss	Everolimus or Temsirolimus, single drug therapy for at least 6 months	Baseline	At least 6 months
Campistol et al. [23]	2005	RCT (secondary analysis)	Human renal transplant recipients	115	Bone metabolism	Sirolimus, dose adjusted to maintain blood trough concentrations at 30 ng//ml for 2 months, 15 ng/ml after 2 months + All received glucocorticosteriods Study A received Azathioprine additionally Study B received Mycophenolate additionally	Cyclosporin A (combination), dose adjusted to maintain blood trough concentrations at 200–400 mg/ml for 2 months, 100–200 ng/ml after 2 months	At least 24 weeks
Westenfeld et al. [24]	2011	Cross-sectional	Human renal transplant recipient (6–195 months after transplantation)	42	Bone metabolism	Triple therapy of steroids + Sirolimus + (Mycophenolate or Azathioprine), trough levels at 5–10 ng/ml	Calcineurin based triple therapy, (cyclosporine trough levels at 80–120 ng/ml, tacrolimus 8–12 ng/ml)	
Sessa et al. [25]	2010	Cohort (prospective)	Human renal transplant recipients	24	Impact on prevalence factors for post-renal transplant osteopathy	Sirolimus (combination with Mycophenolate or steroid)	Multiple groups with different immunosuppressive protocols	
Gnant et al. [26]	2013	RCT (exploratory analysis)	Postmenopausal women with metastatic or locally advanced, oestrogen positive HER2 breast cancer	724	Bone turnover	Everolimus, 10 mg, each day + Exemestane, 25 mg, each day	Placebo control (placebo + exemestane), 25 mg, each day	18 months
Hadji et al. [27]	2018	Non-randomized clinical trial (exploratory analysis)	Postmenopausal women with HR + , HER2-negative locally advanced or metastatic breast cancer	299	Bone turnover	Everolimus, 10 mg, each day + Exemestane, 25 mg, each day	Baseline	48 weeks
Bruyn et al. [34]	2007	RCT	Rheumatoid arthritis patients	121	Rheumatoid arthritis response	Everolimus, 6 mg, each day + Methotrexate + (NSAIDs and prednisone if stable dose)	Placebo control	3 months
Wen et al. [35]	2019	RCT	Rheumatoid arthritis patients	62	Rheumatoid arthritis response, Impact on immune cells	Sirolimus, 0.5 mg, once every two days + (Combination with other immunosuppressants based on participant)	Control (receiving other immunosuppressives)	24 weeks
Niu et al. [36]	2020	Non-randomized clinical trial	Rheumatoid arthritis patients	115	Rheumatoid arthritis response, Impact on immune cells	Sirolimus, 0.5 mg, once every two days + (Combination with other immunosuppressants based on participant)	RA patients under conventional treatment, Healthy volunteers	12 weeks

#### Bone changes

Five studies included the effects of rapamycin and rapalogues on bone as an outcome [23–27]. Campistol et al. [23] investigated the effects of rapamycin on bone metabolism in renal transplant patients, using serum osteocalcin and urinary *N*-telopeptide (i.e., bone-associate collagen degradation) as proxy markers of bone anabolism and catabolism. Reduced serum osteocalcin was observed in participants receiving rapamycin treatment than in those treated with the immunosuppressant cyclosporine A (*P* < 0.001 for weeks 12 and 24, and *P* < 0.008 at week 52). A significant reduction in urinary *N*-telopeptide levels in participants receiving rapamycin was also observed at week 24 (*P* = 0.018), and this remained consistently lower than in participants receiving cyclosporine A.

Westenfeld et al. [24] compared the effects of rapamycin vs. calcineurin inhibitor-based immunosuppression therapy in a cross-sectional study of renal transplant patients. The authors suggested that rapamycin could promote bone health by reducing osteoclast maturation and activity. They used serum TRAP-5b and RANKL as markers of bone metabolism and osteoclast differentiation. Compared to calcineurin inhibitor-based immunosuppression therapy, the study found significantly lower levels of TRAP-5b (*P* < 0.05; and *P* = 0.018 when accounting for confounders), and sRANKL (*P* < 0.05) in those treated with rapamycin. Complementary in vitro data showed rapamycin treatment suppressed osteoclast maturation, confirming that TRAP-5b was a more specific marker of osteoclast activity.

Sessa et al. [25] investigated the impact of various immunosuppressive regimens on different post-transplant renal osteopathy prevalence factors. Mean values of calcitonin (a thyroid hormone for calcium homeostasis) were higher in a group using tacrolimus combination therapy compared with the rapamycin combination group (*P* = 0.048). The results of this study did not otherwise show an effect on bone health that could be attributed to rapamycin. However, this study is conducted on a population of renal transplant patients who have likely had long-term osteodystrophy changes. As noted by the authors, treatment should start early as bone loss occurs just months post-transplantation [25].

Gnant et al. [26] examined the effects of everolimus with exemestane on bone marker levels in postmenopausal women with breast cancer. Overall, additional treatment with everolimus significantly decreased the serum bone markers bone-specific alkaline phosphatase (BSAP), procollagen type 1 N-terminal polypeptide (P1NP), and C-terminal cross-linking telopeptide type 1 collagen (CTX) from baseline, compared to the exemestane-only group (*P* < 0.01) at week 6. This trend generally continued onto the 12th week. Further, those with baseline bone metastases also had lower turnover markers than participants with no baseline bone metastases. The author attributed these findings to the additional anti-cancer effects of everolimus and the suppression of osteoclastogenesis.

Similarly, Hadji et al. [27] also investigated the effects of everolimus and exemestane therapy on bone in postmenopausal women with breast cancer [27]. However, patients were allowed prior/concomitant antiresorptive treatment (ART), and no controls were used. The authors’ conclusions were consistent with Gnant et al. [26] in that everolimus impaired osteoclast maturation and reduced bone turnover [26]. Significant changes were reported for all markers at 24 weeks [27], where mean changes from baseline for procollagen type I N-terminal propeptide (P1NP), osteocalcin, parathyroid hormone (PTH), 25-OH-vitamin D, and CTX were all reduced (*P* < 0.001) except for CTX (*P* = 0.036). ART patients also had a more significant reduction than those not receiving ART. Interestingly, the presence of baseline bone metastases did not meaningfully impact bone marker levels [27], opposing Gnant et al.’s [26] previous findings. This may be due to a few reasons: (1) a difference in the proportion of bone metastases between studies (59.3% in Hadji et al. [27] vs. 76.7% in Gnant et al. [26]), (2) a difference in the baseline bisphosphonate use (24.1% in Hadji et al. [27] vs. 47.2% in Gnant et al. [26]).

#### Skeletal muscle changes

Six studies examined the impact of rapalogues on skeletal muscle health and function as an outcome [28–33]. Gundermann et al. [28], Dickinson et al. [29, 30], and Drummond et al. [31] followed similar trial protocols.

Drummond et al. [31] demonstrated that rapamycin inhibited contraction-induced skeletal muscle protein synthesis (“SKMPS”) vs. the control (*P* < 0.05). Similarly, Gundermann et al. [28] showed that rapamycin inhibited SKMPS in the context of blood flow restriction exercise. Here, SKMPS was unchanged at all the time points for the rapamycin group (*P* < 0.05), whereas the control group demonstrated elevated levels of SKMPS (*P* < 0.05).

In one study, Dickinson et al. [29] observed that rapamycin inhibited L-essential amino acid (EAA) and stimulated SKMPS (*P* < 0.05). In a separate study, the same team [30] analyzed rapamycin’s effect on post-absorptive SKMPS or breakdown. Basal skeletal muscle protein metabolism changes were reported as insignificant following short-term administration of rapamycin (*P* > 0.05), concluding that rapamycin may only inhibit muscle synthesis in the presence of stimuli such as mechanical contractions or increased levels of EEAs.

Veasey-Rodrigues et al. [32] performed a pilot study analyzing body composition changes over 8 weeks from using Temsirolimus in patients with advanced solid tumours. Results show that there were no significant changes in body composition from baseline, such as skeletal muscle area (*P* = 0.57), skeletal muscle index (SMI) (*P* = 0.36) and lean body mass (LBM) (*P* = 0.56).

Gyawali et al. [33] conducted a retrospective study investigating the effect of long-term everolimus or temsirolimus use (> 6 months) in renal/pancreatic cancer patients. Long-term use of these rapalogues decreased skeletal muscle tissue (SMT) (*P* = 0.011), SMI (*P* = 0.022), and LBM (*P* = 0.007). However, changes in body weight were insignificant (*P* = 0.721). In this case, the explanation for the skeletal muscle loss was cachexia. However, the authors noted a 6-month use of rapalogues as an inclusion criterion, allowing the change in muscle mass to be reliably associated with the rapalogues instead of cancer cachexia. Interestingly, as their study population involved cancer patients, the following mechanisms of muscle wasting were relevant: elevated cytokines, reduced physical activity, and altered metabolism.

#### Rheumatoid arthritis

Three studies were identified [34–36], which examined the effects of rapamycin and rapalogues on RA. Bruyn et al. [34] investigated whether combinatorial treatment with everolimus and methotrexate could improve outcomes [34]. At 12 weeks, the everolimus and methotrexate combination group had a better response through the ACR20 (a criterion used to determine RA improvement) assessment (*P* = 0.022). The patient’s assessment of disease activity showed everolimus responded better (*P* = 0.004), and the clinical response compared to baseline was also significant (*P* = 0.024).

Wen et al. [35] studied the effects of low dose rapamycin on disease activity and immunological cells in patients with RA. Participants administered rapamycin were allowed to use other immunosuppressants. The results illustrated a clinical improvement through the decrease in the DAS28-ESR (an assessment of RA severity) score [37] from week 3 to week 24 (*P* < 0.001) from baseline. However, this decrease was insignificant compared to the control group on conventional treatment. The study acknowledged that participants on disease-modifying anti-rheumatic medications (DMARDs) had reduced the doses if given rapamycin. Moreover, the number of regulatory T cells (Treg) was higher compared to the conventional group (*P* < 0.05) at week 24. Wen et al. [35] provide insight into why anti-inflammatory Treg cells may improve RA in the long term.

Likewise, Niu et al. [36] explored the use of low dose rapamycin in low disease activity RA patients to determine its effect on Treg and other immune cells. The authors' deduction of why improvements in Treg levels can be attributed to the addition of rapamycin was consistent with Wen et al. [35]. At 12 weeks, Niu et al. [36] showed an increase in absolute count of the number of Treg cells in the rapamycin treatment group (*P* = 0.013) and percentage (*P* = 0.02) compared to baseline. Pro-inflammatory T-helper cell 17 (Th17) to anti-inflammatory Treg ratios were also significantly improved from baseline for those administered Rapamycin (*P* = 0.005), which was not the case in the conventional treatment group (*P* = 0.655). For the rapamycin treatment group, the clinical response was positive but not significant by the end of the study (DAS28 2.25 vs. 2.53 at the beginning). Furthermore, rapamycin treatment had a higher number of patients that were in DAS28 remission (DAS28 < 2.6) compared to baseline (71.4%). Those that achieved or remained in remission also had higher Treg levels.

The studies included in this review report adverse effects associated with using rapalogues in humans. A summary of the key adverse events and safety issues is listed in Table 4.

**Table 4:** Key toxicity profiles and adverse events

Study	Key adverse events
Gundermann et al. [28] Activation of mTORC1 signaling and protein synthesis in human muscle following blood flow restriction exercise is inhibited by rapamycin	No issues raised - Study takes place over a short period and in healthy, young volunteers
Dickinson et al. [29] Mammalian target of rapamycin complex 1 activation is required for the stimulation of human skeletal muscle protein synthesis by essential amino acids	No issues raised - Study takes place over a short period and in healthy, young volunteers
Dickinson et al. [30] Rapamycin does not affect post-absorptive protein metabolism in human skeletal muscle	No issues raised - Study takes place over a short period and in healthy, young volunteers
Drummond et al. [31] Rapamycin administration in humans blocks the contraction-induced increase in skeletal muscle protein synthesis	No issues raised - Study takes place over a short period and in healthy, young volunteers
Veasey-Rodrigues et al. [32] A pilot study of Temsirolimus and body composition	Toxicity data—Study listed most common toxicities that were, at least possibly, drug-related. These were grouped into 2 groups: grade 1–2 and grade 3–4, based on the Common Terminology Criteria for Adverse Events version 3.0 Grade 1–2 - Fatigue (100%) - Anaemia (100%) - Hyperglycaemia (81%) - Hypercholesterolemia (75%) Grade 3–4: - Anaemia - Thrombocytopenia - Leukopenia/neutropenia - Aspartate aminotransferase/alanine aminotransferase elevations The study did not correlate baseline sarcopenia with a toxicity profile - Median number of toxicities per patient was 7 between sarcopenic and non-sarcopenic participants Study noted no significant difference between toxicities in participants and their baseline body composition
Gyawali et al. [33] Muscle wasting associated with the long-term use of mTOR inhibitors	The Time to treatment failure (TTF) was another outcome measured in the study. The author explains its definition as the time between starting the mTOR inhibitor to when it is stopped The author lists reasons for stopping as adverse events, disease progression, or mortality - However, there currently is no further detail into the reasons for the participants stopping treatment TTF was noted not to be associated with the sarcopenic status of patients
Campistol et al. [23] Bone metabolism in renal transplant patients treated with cyclosporine or sirolimus	Safety information was obtained from individual studies analyzed by Campistol et al *Study 1* Participants that discontinued the study due to several reasons. Notable ones possibly relating to the use of the mTOR inhibitor include: Leukopenia - Thrombocytopenia - Hypercholesterolemia/hyperlipidaemia - Increased liver enzymes Key adverse events (sirolimus vs cyclosporin A) - Hypertriglyceridemia (51% vs 12%, *P* < 0.01) - Hypercholesterolemia (44% vs 14%, *P* < 0.01) - Hyperglycaemia (20% vs 7%) - Insulin dependent diabetes (2% vs 2%) - SGOT (aspartate aminotransferase) elevation (17% vs 0, *P* < 0.05) - Hypokalaemia (34% vs 0, *P* < 0.01) - Hypophosphatasaemia (15% vs 0, *P* < 0.05) - Thrombocytopenia (37% vs 0, *P* < 0.01) - Leukopenia (39% vs 14%, *P* < 0.05) - Anaemia (37% vs 24%) - Arthralgia (20% vs 0, *P* < 0.05) - Pneumonia (17% vs 2%, *P* < 0.05) Sirolimus group also experienced a higher number of infections, but numbers were still similar (*n* = 25 vs *n* = 22) *Study 2* Some discontinuations of participants in the sirolimus group were due to agranulocytosis and hyperlipidaemia Key adverse events (sirolimus vs cyclosporin A) - Hypertriglyceridemia (73% vs 50%) - Hypercholesterolemia (65% vs 45%) - Hyperglycaemia (15% vs 16%) - Insulin dependent diabetes (3% vs 3%) - SGOT (aspartate aminotransferase) elevation (13% vs 5%) - Creatinine increase (18% vs 39%, *P* < 0.05) - Hyperuricemia (3% vs 18%, *P* < 0.05) - Thrombocytopenia (45% vs 8%, *P* < 0.01) - Leukopenia (28% vs 18%) - Anaemia (43% vs 29%) - Diarrhoea (38% vs 11%, *P* < 0.01)
Westenfeld et al. [24] Impact of sirolimus, tacrolimus and mycophenolate mofetil on osteoclastogenesis—implications for post-transplantation bone disease	Key metabolic effects in sirolimus vs calcineurin inhibitor - Cholesterol (Elevated, *P* = 0.001) - Triglycerides (Elevated, *P* = 0.002) - Haemoglobin (Decreased, *P* = 0.048) - Intact parathyroid hormone (Elevated, *P* = 0.032) Study explained no change in platelets or leucocyte count between groups
Sessa et al. [25] Immunosuppressive agents and bone disease in renal transplant patients with hypercalcemia	No information
Gnant et al. [26] Effect of Everolimus on Bone Marker Levels and Progressive Disease in Bone in BOLERO-2	Safety information obtained from poster used in San Antonio Breast Cancer Symposium, as referenced by Gnant et al Study graded adverse events from 1 to 4 Key adverse events at all grades (everolimus vs placebo) - Hyperglycaemia (14% vs 1%) - Pneumonitis (16% vs 0) - Stomatitis (59% vs 12%) - Rash (39% vs 7%) - Fatigue (37% vs 27%) - Diarrhoea (34% vs 19%) - Nausea (31% vs 29%) - Decreased weight (28% vs 7%) Most common grade 3–4 adverse events (everolimus vs placebo) - Stomatitis (8% vs < 1%) - Hyperglycaemia (5% vs < 1%) - Fatigue (4% vs 1%) Bone-related adverse events were reported to be low and similar across treatment arms, though fewer fractures were reported in the everolimus arm (2.3% vs 3.8%) The population in the everolimus arm was twice that in the placebo arm (*n* = 428 vs *n* = 238)
Hadji et al. [27] The impact of mammalian target of rapamycin inhibition on bone health in postmenopausal women with hormone receptor-positive advanced breast cancer receiving everolimus plus exemestane in the phase IIIb 4EVER trial	In the study, 24.7% of discontinuations were due to adverse events. 53.7% of patients required at least one dose reduction of everolimus Key common adverse events in safety population (all grades, grade 3–4) - Stomatitis (49.2%, 8.4%) - Fatigue (36.1%, 3.3%) - Diarrhoea (26.4%, 2%) - Nausea (26.1%, 3%) - Rash (22.7%, 1%) - Anaemia (17.7%, 4.3%) - Thrombocytopenia (7.7%, 1.7%) - Hyperglycaemia (5%, 1.3%) Skeletal adverse events - Fracture (2.7%) - Osteonecrosis of the jaw (0.7%) - Osteoporosis (0.3%)
Bryun et al. [34] Everolimus in patients with rheumatoid arthritis receiving concomitant methotrexate: a 3-month, double-blind, randomized, placebo-controlled, parallel-group, proof-of-concept study	88.5% of the everolimus arm population reported adverse events compared to the 70% reported in the placebo arm Slightly more participants in the everolimus arm discontinued due to adverse (10 vs 6) Key drug-related adverse events at 12 weeks (everolimus vs placebo) - Gastrointestinal disorders (31.1% vs 10%) - Skin/subcutaneous disorders (16.4% vs 1.7%) - Infections/infestations (9.8% vs 1.7%) - Hypercholesterolemia (6.6% vs 1.7%) An increase in lipids was noted in the everolimus group to be statistically significant but returned to baseline at the end of the treatment. More of the everolimus population had exceeded the upper limit in tests compared to the placebo Leucocyte/neutrophil count was decreased significantly in the everolimus group at week 12 from baseline but returned to baseline by 24 weeks Platelets were also decreased, but the difference was not significant The study mentions that liver markers (AST, ALT, alkaline phosphatase) were raised in the everolimus arm but not considered clinically meaningful
Wen et al. [35] Low-dose sirolimus immunoregulation therapy in patients with active rheumatoid arthritis: a 24-week follow-up of the randomized, open-label, parallel-controlled trial	One patient was known to have discontinued study due to lower limb oedema, attributed to sirolimus intolerance Key safety outcomes - Red blood cell counts and haemoglobin concentration had no significant changes compared to control (P > 0.05) - Platelet count was significantly higher in sirolimus arm at one time point (3 weeks) but insignificant at others - Neutrophilic granulocyte levels were lower compared to baseline at weeks 3 and 12 (P < 0.05) - Liver function markers were not affected - Renal function was not affected No cases of thrombocytopenia, mucositis, or proteinuria were observed
Niu et al. [36] Sirolimus selectively increases circulating Treg cell numbers and restores the Th17/Treg balance in rheumatoid arthritis patients with low disease activity or in DAS28 remission who previously received conventional disease-modifying anti-rheumatic drugs	Study explains no serious adverse events occurred during the study Minor adverse events include - Rash - Oral ulcers - Alopecia These side effects occurred in 3 participants and did not require special care

## Discussion

To our knowledge, this is the first systematic review on the effect of rapamycin and rapalogues on MSKD in humans. Although these novel therapeutic approaches have been tested in humans and have shown beneficial effects on MSKD, mainly bone and joints, the evidence supporting their use in humans for these conditions is still limited.

Regarding bone health, most studies showed that rapamycin and rapalogues positively regulate bone turnover via a reduction in osteoclastogenesis [23, 24, 26, 27]. However, it is crucial to consider that the clinical context in which these interventions were evaluated does not correspond merely to aging. Among the studied populations, osteoporosis risk factors were highly prevalent: chronic steroid use, low oestrogen, low vitamin D, and immobilization [38], and many of these factors may contribute as confounding, such as renal osteodystrophy, post-transplant hyperparathyroidism, and increased bone resorption associated with aromatase inhibitor therapy and bone metastases [39, 40]. Finally, although the reduction in osteoclastogenesis is a promising outcome, these studies would ideally include more relevant clinical outcomes such as bone density by DXA scans [41] to quantify rapamycin’s effects on bone density.

In opposition to what was found in basic studies where an anabolic effect of rapalogues on muscle mass was observed, three studies [29–31] showed negative effects of rapamycin and rapalogues on skeletal muscle metabolism. However, it is important to analyze the population studied and the protocol with which the intervention was implemented. The participants in these studies were young, healthy participants, and the intervention was short in time. As mTORC1 activity increases in aging and contributes to muscle loss [18], extrapolating the short-term effects of rapamycin to long-term outcomes of muscle maintenance/loss is too speculative. Furthermore, skeletal muscle composition differs significantly in aged individuals due to long term changes in muscle mass, reduced hormone synthesis (e.g., growth hormone, oestrogen), the presence of inflammatory cytokines and adipokines, and decreased physical activity [6]. While our review aims to comment on rapamycin and rapalogues for use in an aged population, it is still informative to observe how rapamycin and rapalogues affect healthy muscle as a rationale for future clinical trials in older participants. Longer-term studies of rapamycin treatment included cancer patients [32, 33]. This introduces different muscle wasting pathologies: MSKD and disuse, vs. chronic disease and muscle atrophy, or both [42, 43]. One study proposed muscle loss from long-term rapamycin use was likely due to chronic mTORC1 inhibition [33]; however, the lack of a control group and short follow-up time limit these findings. Notably, these studies [32, 33] involved participants with median ages more representative of an aging population, i.e., when age-related muscle pathology and the gradual nature of sarcopenia can manifest [44].

In RA, rapamycin improved biochemical and clinical outcomes of patients receiving methotrexate while having comparable side effects [34]. This may be due to both rapamycin and methotrexate targeting cell proliferation pathways. This is evidenced by Wen et al. [35], who observed decreased DMARD intake when rapamycin was added to the therapeutic regimen. Future studies should confirm the safety and efficacy of combinatorial rapamycin (or rapalogues) and DMARD therapies.

The toxicity profile of rapamycin is different in humans than in animal models and is an important point to discuss. Common adverse reactions in humans include immunosuppression, oral ulcers hyperglycaemia/diabetes, hyperlipidaemia and hypercholesterolemia [12]. Mannick et al. [45] reported on everolimus and concluded it was safe among subjects over 65 years. Similarly, Kraig et al. [46] reported that 8 weeks of rapamycin was safely tolerated by subjects 70–95 years otherwise healthy. However, there were trends for increased HbA1c and cholesterol [46] and similar views on side effects were shared in the studies included in this systematic review. Short-term rapamycin administration and/or low doses of the drug did not produce any major concerns over side effects [28–31, 35, 36]. However, longer-term studies showed increased metabolic disturbances such as hyperlipidaemia and hyperglycaemia [23, 24, 26, 27, 32, 34]. These metabolic consequences [47, 48] may be an issue in patients treated with rapamycin, who also present with cardiovascular disease (or risk factors thereof). Overall, while rapamycin is generally safe, further studies are required to ensure common side effects can be safely managed. Future studies should also determine the “optimal dose” of rapamycin and rapalogues that produce the desired effects.

Limitations of this systematic review include the low number of studies and the diverse study designs. There are differing characteristics between study participants (e.g., young age) and the population of interest (e.g., aging population). This systematic review aimed to broadly appraise current literature involving human participants by having inclusion criteria encompassing multiple study types, meaning different levels of evidence were included, and exposure to other biases was possible. Such study types comprise non-randomized trials, non-blinded studies, cross-sectional studies, cohort studies, single-arm trials, and secondary analyses of other trials. Other issues with some study designs would be the smaller sample sizes, different dosages of the drug, co-interventions varying between groups and shorter follow-up duration.

## Conclusion

This is the first study looking at the effect of rapamycin and rapalogues on MSKD. Studies included in this review have mostly evaluated the effect of rapamycin and rapalogues on MSKD in humans, showing anabolic effects on bone metabolism and reducing the inflammatory activity of RA. However, the evidence supporting its use is still incipient, and the clinical implication of these results on the management of osteoporosis, sarcopenia, or osteosarcopenia has not been studied, opening an interesting field for future research.

## Data Availability

Data available on request from the authors.
